# Incidence and risk factors of postoperative acute myocardial injury in noncardiac patients: A systematic review and meta-analysis

**DOI:** 10.1371/journal.pone.0286431

**Published:** 2023-06-15

**Authors:** Yuan Chang, Mengjiao Zhou, Jing Huang, Yanqiong Wang, Jianlin Shao

**Affiliations:** Department of Anesthesiology, the First Affiliated Hospital of Kunming Medical University, Kunming, China; Wiltse Memorial Hospital, REPUBLIC OF KOREA

## Abstract

**Introduction:**

Postoperative myocardial injury after noncardiac surgery is common and is associated with short- and long-term morbidity and mortality. However, the incidence and risk factors for postoperative acute myocardial injury (POAMI) are currently unknown due to inconsistent definitions.

**Methods:**

We systematically searched PubMed and Web of Science to identify studies that applied the change value of preoperative and postoperative cardiac troponins to define cardiac injury. We estimated the pooled incidence, risk factors, and 30-day and long-term mortality of POAMI in noncardiac patients. The study protocol was registered with PROSPERO, CRD42023401607.

**Results:**

Ten cohorts containing 11,494 patients were included for analysis. The pooled incidence of POAMI was 20% (95% CI: 16% to 23%). Preoperative hypertension (OR: 1.47; 95% CI: 1.30 to 1.66), cardiac failure (OR: 2.63; 95% CI: 2.01 to 3.44), renal impairment (OR: 1.66; 95% CI: 1.48 to 1.86), diabetes (OR: 1.43; 95% CI: 1.27 to 1.61), and preoperative beta-blocker intake (OR: 1.65; 95% CI: 1.10 to 2.49) were the risk factors for POAMI. Age (mean difference: 2.08 years; 95% CI: -0.47 to 4.62), sex (male, OR: 1.16; 95% CI: 0.77 to 1.76), body mass index (mean difference: 0.35; 95% CI: -0.86 to 1.57), preoperative coronary artery disease (OR: 2.10; 95% CI: 0.85 to 5.21), stroke (OR: 0.90; 95% CI: 0.50 to 1.59) and preoperative statins intake (OR: 0.65; 95% CI: 0.21 to 2.02) were not associated with POAMI. Patients with POAMI had higher preoperative hsTnT levels (mean difference: 5.92 ng/L; 95% CI: 4.17 to 7.67) and lower preoperative hemoglobin levels (mean difference: -1.29 g/dL; 95% CI: -1.43 to -1.15) than patients without.

**Conclusion:**

Based on this meta-analysis, approximately 1 in 5 of noncardiac patients develop POAMI. However, the lack of a universally recognized definition for POAMI, which incorporates diverse cardiac biomarkers and patient groups, poses a challenge in accurately characterizing its incidence, risk factors, and clinical outcomes.

## Introduction

More than 300 million patients worldwide yearly undergo major surgical procedures [1]. After noncardiac surgery, myocardial injury occurs in approximately 20% of patients and is associated with short-term and long-term morbidity and mortality [2–8]. According to the Fourth Universal Definition of Myocardial Infarction (2018), myocardial injury is defined as at least one value of cardiac troponins (cTn) above the 99th percentile upper reference limit of the healthy population, while the acute myocardial injury with a rise and/or fall pattern over a period [9].

The precise etiology behind postoperative myocardial injury is not yet fully understood [8, 10, 11]. The postoperative elevated cTn could be attributed to two potential causes: preoperative chronic myocardial injury, and acute myocardial injury occurring during the surgical procedure and postoperative recovery period. Chronic myocardial injury affects more than 6% of the general population and may be more prevalent in noncardiac patients [12]. Over 40% of patients with cardiovascular diseases have preoperative myocardial injury prior to noncardiac surgery [13–15]. Distinguishing acute myocardial injury from chronic myocardial injury through pre-and postoperative cardiac biomarkers may facilitate the identification of perioperative risk factors that damage cardiomyocytes after anesthesia induction and provide a theoretical basis for future interventional trials.

Despite the recommendation in the Fourth Universal Definition of Myocardial Infarction (2018) to diagnose acute myocardial injury based on significant changes in cardiac troponin concentrations, a clear definition for perioperative acute myocardial injury in noncardiac surgical patients is currently lacking. [16, 17]. From increased 5 ng/L to increased 100% from the preoperative high sensitivity troponin T (hsTnT), the incidence of postoperative acute myocardial injury (POAMI) may vary from 17% to 34% [18]. In this systematic review and meta-analysis, we primarily aimed to investigate the pooled incidence of POAMI in noncardiac patients. With available studies, we further analyzed risk factors, and 30-day and long-term mortality associated with POAMI.

## Methods

This study is a systematic review and meta-analysis of observational studies on adult patients undergoing noncardiac surgery. This review was preregistered in the PROSPERO international prospective systematic review register (CRD42023401607) and performed according to PRISMA guidelines (S1 Checklist) [19]. The primary aim was to evaluate the pooled incidence of POAMI in patients following noncardiac surgery. The secondary aims were to evaluate the association of preoperative risk factors with POAMI and the effect of POAMI on postoperative 30-day and long-term mortality. Long-term follow-up is defined as a follow-up to at least six months after surgery.

### Literature search

A comprehensive and systematic literature search was performed through PubMed and Web of Science to identify relevant studies published before February 1st, 2023. The search was performed using the key terms of "troponin," "myocardial injury," "perioperative," and "postoperative," and was limited to English language publications. The detailed search strategies are listed in S1 Table. Two independent researchers (YC and MZ) carried out the search process, with any discrepancies resolved through a group meeting with a third investigator (JS). To ensure completeness, the reference lists of the included studies were meticulously reviewed, and a citation search was also conducted on all eligible studies.

### Inclusion and exclusion criteria

The inclusion criteria for this study included observational studies (retrospective or prospective), that evaluated changes in cTn levels before and within 30 days after noncardiac surgery in adults who were at least 18 years of age. Exclusion criteria were the following: 1) articles written in languages other than English; 2) reviews, comments, protocols, editorials, letters, case reports, or animal trials; 3) studies with cardiac surgeries; 4) studies applied other cardiac biomarkers than cTn to define myocardial injury; and 5) sample sizes less than 20 cases. If multiple studies from the same cohort were identified, the most recent study was selected for analysis.

### Data extraction, outcomes, and risk-of-bias assessment

The following information was extracted from each included study: name of the first author, year of publication, country, preoperative patient characteristics, the definition of POAMI, and the number of patients with and without POAMI. For case-control studies that reported both unpaired and paired results, we utilized the unpaired results for the analysis of incidence and risk factors of POAMI, and the paired results for the association of POAMI on 30-day mortality and/or long-term mortality. The raw data extracted from each study are available in Table 1.

**Table 1:** Overview of included studies.

First Author, published year, country	Stud design	Sample size	Participants	Procedure	Mean age, years	Male, n (%)	Type of cTnT assay; Low limit of detection; 99th percentile URL	Sampling procedure	Definitions of postoperative acute myocardial injury	Rationale for choosing this changed value to define postoperative acute myocardial injury	Incidence of postoperative acute myocardial injury, n (%)
Chew et al., 2002, Sweden [26]	Prospective cohort	1291	Aged ≥50 years undergoing elective, major abdominal surgery and requiring at least one overnight hospital stay.	Mix	70	699 (54%)	hsTnT; 3 ng/L; 14 ng/L.	Up to 24 h before surgery, after surgery at the PACU, and on days 1, 2, and 3 after surgery or until discharge.	An absolute hsTnT change of 14 ng/L above preoperative level.	Based on the definition of a prospective cohort study [14]	144 (11%)
Gualandro et al., 2021, Switzerland [22]	Prospective cohort	8659	Age 65 to 85 years or age above 45 years in the presence of established coronary artery disease, peripheral artery disease or cerebrovascular disease.	Mix	78	1755 (56%)	hsTnT; NR; 14 ng/L.	Before surgery and on postoperative days 1 and 2.	An absolute change of 14 ng/L above preoperative level (or between two postoperative levels if the preoperative value was missing).	Patients meeting this definition always meet the definition of myocardial injury, regardless of whether their preoperative hsTnT level over 99th URL	1392 (16%)
Sanders et al., 2021, Australia [33]	Prospective cohort	94	Adults undergoing major elective non-intracranial, noncardiac surgery in general anesthesia and requiring at least 2 day of hospital stay.	Mix	NR	54 (57%)	TnI; NR; NR.	Before surgery and on postoperative days 1 to 4.	An absolute TnI change of 40 ng/L above preoperative level.	Based on the results of the VISION study [2]	23 (24%)
Gillmann et al., 2020, Germany [27]	Prospective cohort	118	Adults undergoing an elective open vascular procedure under general anesthesia.	Vascular	70	88 (75%)	hsTnT; NR; 14 ng/L.	Before surgery and on postoperative days 1.	Postoperative hsTnT above 20 ng/L with 5 ng/L elevation from the preoperative level.	Based on the definition of the VISION study [13]	23 (19%)
Kamber et al., 2018, Switzerland [28]	Prospective cohort	30	Adults undergoing higher risk vascular surgery.	Vascular	70	24 (80%)	hsTnT; NR; 14 ng/L.	Before surgery, immediately after surgery (0 h), 2, 4, 6, 8 h after surgery, and on the first and second postoperative day.	Postoperative hsTnT above 20 ng/L with 5 ng/L elevation from the preoperative level.	Based on the definition of the VISION study [13]	6 (20%)
Szczeklik et al., 2018, Poland [29]	Prospective cohort	239	Aged ≥ 45 years and stayed at least overnight in hospital after the endovascular revascularization procedure.	Vascular	72	134 (56%)	hsTnT; NR; 14 ng/L.	Before, 3 to 6 hours after surgery and postoperative day 1.	Postoperative hsTnT above14 ng/L with 30% relative change from the preoperative level.	NA	61 (28%)
Toda et al., 2018, Japan [30]	Prospective cohort	151	Aged ≥60 years with preserved EF undergoing elective non-cardiac surgery.	Mix	74	66 (57%)	hsTnT; NR; 14 ng/L.	Before and postoperative day 1 and 3.	Postoperative hsTnT above14 ng/L and 20% relative change from the preoperative level.	From previous studies [18, 27, 34] and professional opinions [35]	36 (24%)
Górka et al. 2017, Canada [31]	Nested case–control	475	Aged >45 years underwent elective surgery for peripheral artery disease or an abdominal aortic aneurysm.	Vascular	NR	NR	hsTnT; NR; 14 ng/L.	Before surgery, 6–12 h after surgery, and on postoperative day 1 to 3.	Postoperative hsTnT above 50 ng/L with 20% increase from the preoperative level.	From the VISION study [2] and professional opinions [36, 37]	47 (10%)
Thomas et al., 2016, New Zealand [32]	Prospective cohort	85	Adults undergoing elective major vascular procedure.	Vascular	74	61 (72)	hsTnT; NR; 14 ng/L.	Preoperative and postoperative 6, 12, 24, 48, and 72 hours.	A change of 50% with at least one value 14 ng/L and a change of 20% when the preoperative level was 53 ng/L.	From professional opinions [37] and a guideline [38]	35 (41%)
Alcock et al. 2012, Australia [18]	Prospective cohort	352	Aged >45 years undergoing major elective noncardiac surgery and receiving antiplatelet therapy.	Mix	72	226 (64)	hsTnT; 5 ng/L; 13 ng/L.	Before and postoperative day 1 and 2.	Postoperative hsTnT above 14 ng/L with 50% increase from the preoperative level.	From a guideline [39].	79 (22%)

cTnT: cardiac troponins; hsTnT: high-sensitivity troponin T; cTnI: cardiac troponin I; PACU: postoperative care unit; URL: upper reference limit; VISION study: the Vascular events In non-cardiac Surgery patIents cOhort evaluatioN (VISION) study, a large international prospective cohort study; NR: not reported.

### Quality assessment

The quality and potential risk of bias of the observational studies were evaluated using the Newcastle-Ottawa Quality Assessment Scale (NOS) and listed in S2 Table by two investigators (YC and MZ) [20]. The NOS consists of three dimensions: selection, comparability, and exposure, each of which is assessed by 8 items. A score of 4 points can be assigned for selection, 2 points for comparability, and 3 points for exposure, with a total possible score of 9 points. Studies with scores ranging from 1 to 3 were considered to have low quality, those with scores between 4 and 6 were considered to have intermediate quality, and those with scores greater than 7 were considered to have high quality.

### Statistical analysis

The present study conducted the meta-analysis using Review Manager 5.3 (Cochrane Collaboration, Oxford, UK). The summary estimates of the included studies were calculated using the inverse variance method with a random-effects model (DerSimonian-Laird estimator) and presented with 95% confidence intervals [21]. We used the Higgins I-squared (I^2^) index to evaluate the potential heterogeneity. I^2^ values of 0%, 25%, 50%, and 75% represented the thresholds for no, low, moderate, and high heterogeneity, respectively. Forest plots were used to display the results visually, and funnel plots were employed to assess publication bias if at least ten studies reported the same outcome.

The incidence of POAMI was calculated by pooling the incidence and associated 95% confidence intervals from included studies. If studies applied both cardiac troponin T (cTnT) and cardiac troponin I (TnI) to define myocardial injury, we used the results of cTnT assays to synthesize the pooled result. The same perioperative characteristics reported in at least three studies were included to investigate risk factors of POAMI. Continuous and dichotomous perioperative risk factors were analyzed using the weighted mean differences and odds ratios (OR), respectively. The effect of each included study on 30-day and long-term mortality was assessed using hazard ratios (HR). The present investigation involved the extraction of adjusted hazard ratios from the relevant studies. If only unadjusted hazard ratios were obtainable, they were still extracted, and a sensitivity analysis was subsequently conducted to assess their impact.

Subgroup analyses were pre-planned to explore the effects of different surgeries, different troponin assays, and different POAMI definitions with absolute or relative cTn changes on the pooled incidence of POAMI. Additionally, sensitivity analyses were carried out to determine the robustness of the results by excluding studies that utilized the absolute or relative changes of cTn within postoperative days to define POAMI.

## Results

### Description of studies

**Fig 1:**
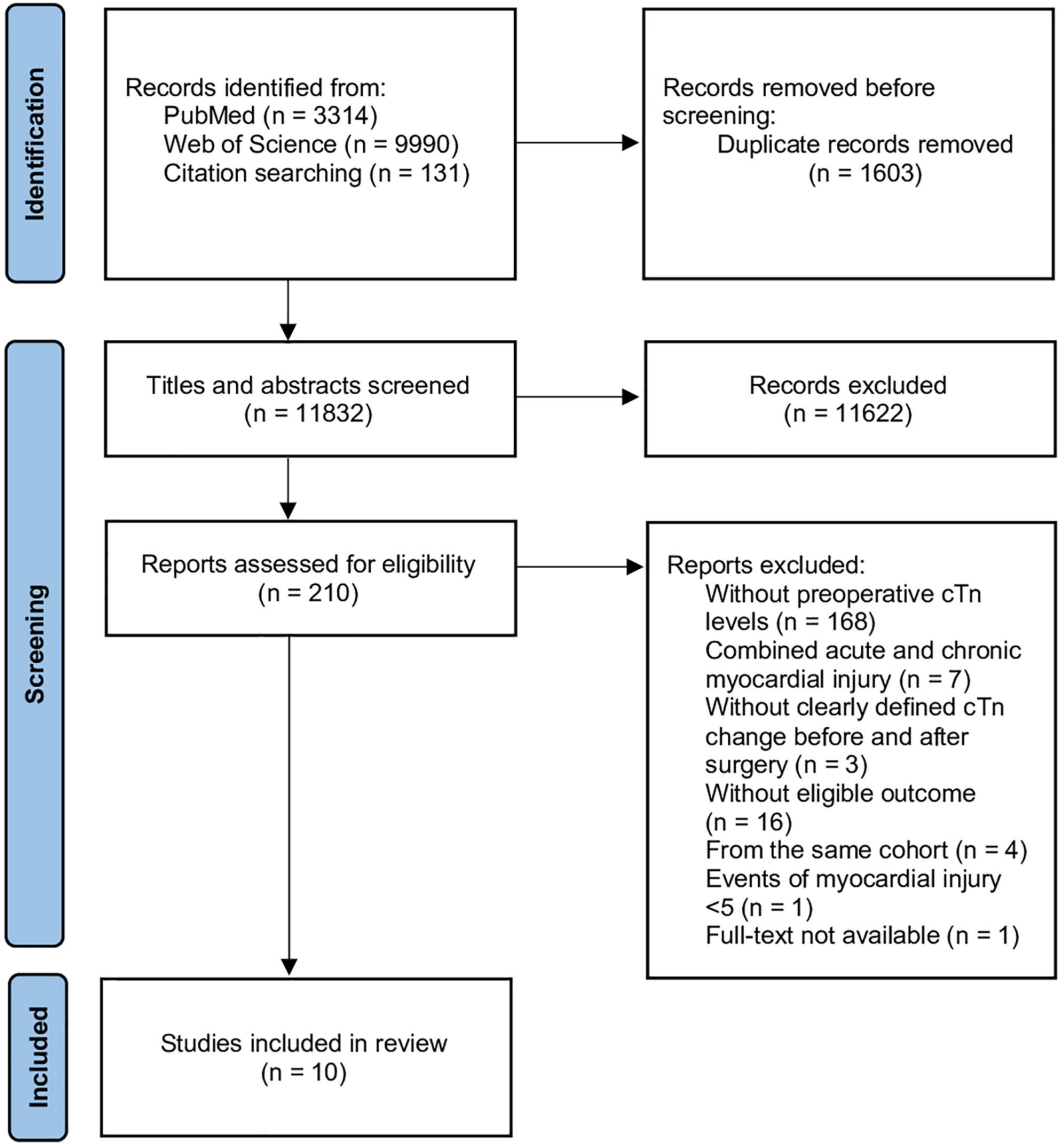
PRISMA flow chart depicting the screening process.

The initial literature search identified 13,435 studies, which were reduced to 1603 after the removal of duplicates. After the screening of titles and abstracts, 210 articles were retrieved for full-text evaluation. 80% (168/210) of studies that reported postoperative myocardial injury did not consider the preoperative level of cardiac troponins. Fifteen studies fulfilled the inclusion criteria and five of them are from the same observational cohort [14, 22–25], and we chose the most recently published one [22]. Finally, 10 studies were included in the analysis with a total sample size of 11,494 noncardiac patients (ranging from 30 to 8659) (Fig 1).

Among ten studies, nine were prospective cohorts and one was a nested case-control study. CTn assays were systematically performed before and within 2 to 3 days after surgery in all 10 studies. Eight studies applied hsTnT [18, 26–32], one study applied TnI [33], and one study applied both hsTnT and TnI [22]. Study and patient characteristics are summarized in Table 1.

### Quality assessment

In total, one study had an intermediate quality and nine had a high quality (S2 Table). The key domains of measurement of intervention, measurement of data, and selection of reported results were judged as low risk of bias in most studies. A high risk of bias due to deviation from the patient selection was found in eight studies.

### Definitions of postoperative acute myocardial injury

Among the ten articles that were included in this analysis, a total of eight different definitions of POAMI were identified (Table 1). These definitions were developed based on either the absolute [22, 26–28, 33] or relative [18, 29–32] difference between preoperative and postoperative troponin levels, and were used either in isolation [22, 26–28, 33] or in combination [18, 29, 30, 32]with an assay-specific 99th upper reference level (URL). The rationale for selecting a particular change value between pre- and post-surgical troponin concentration to determine POAMI varied among the studies, with some being based on expert opinion or guidelines [18, 30–32], others on the characteristics of cardiac troponin assays [22, 31, 32], and still others on results from prior observational studies [26–28, 30, 31, 33]. One study did not provide the rationale for the choice of a specific delta hsTnT level [29].

### Incidence of postoperative acute myocardial injury

**Fig 2:**
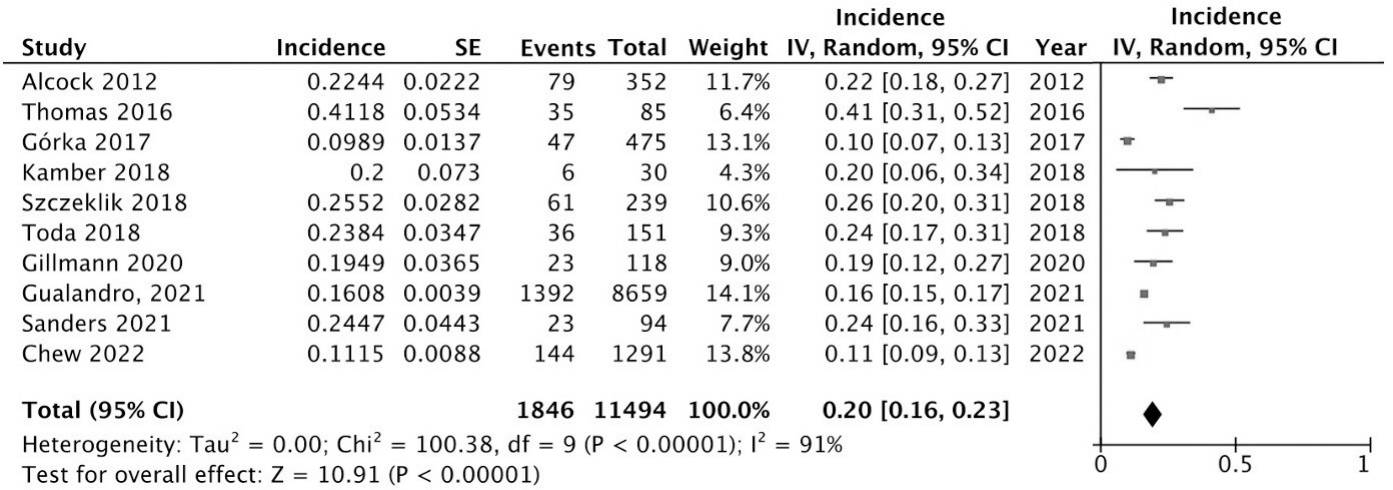
Pooled incidence of postoperative acute myocardial injury.

The incidence of POAMI ranged from 10% to 41% across the included studies. The random effects meta-analysis estimated that the pooled incidence of POAMI was 20% (95% CI: 16% to 23%, I^2^ = 91%) (Fig 2).

### Risk factors of postoperative acute myocardial injury

A total of 13 risk factors were eligible for meta-analysis: preoperative characteristics (age, sex, body mass index (BMI), coronary artery disease, hypertension, cardiac failure, diabetes, stroke/transient ischemic attack (TIA), renal impairment, preoperative medications (statins, beta-blockers); preoperative laboratory testing (hemoglobin, hsTnT). We standardized the measurements of age in years, BMI in kg/m^2^, hemoglobin in g/dL, and hsTnT in ng/L. The identification of preoperative risk factors, such as coronary artery disease, hypertension, cardiac failure, diabetes, stroke/TIA, and renal impairment, was based on the definitions used in the studies that were included in this review.

Patients with POAMI were more likely to have preoperative hypertension (OR: 1.47; 95% CI: 1.30 to 1.66; I^2^ = 0%) (S1 Fig), cardiac failure (OR: 2.63; 95% CI: 2.01 to 3.44; I^2^ = 15%) (S2 Fig), renal impairment (OR: 1.66; 95% CI: 1.48 to 1.86; I^2^ = 0%) (S3 Fig), diabetes (OR: 1.43; 95% CI: 1.27 to 1.61; I^2^ = 0%) (S4 Fig), and preoperative beta-blocker intake (OR: 1.65; 95% CI: 1.10 to 2.49; I^2^ = 48%) (S5 Fig) than patients without (Table 2). Age (mean difference: 2.08 years; 95% CI: -0.47 to 4.62; I^2^ = 81%) (S6 Fig), sex (male, OR: 1.16; 95% CI: 0.77 to 1.76; I^2^ = 54%) (S7 Fig), BMI (mean difference: 0.35; 95% CI: -0.86 to 1.57; I^2^ = 45%) (S8 Fig), coronary artery disease (OR: 2.10; 95% CI: 0.85 to 5.21; I^2^ = 98%) (S9 Fig), stroke/TIA (OR: 0.90; 95% CI: 0.50 to 1.59; I^2^ = 61%) (S10 Fig), and preoperative statins intake (OR: 0.65; 95% CI: 0.21 to 2.02; I^2^ = 89%) (S11 Fig) was not associated with POAMI (Table 2). Patients with POAMI had higher preoperative hsTnT levels (mean difference: 5.92 ng/L; 95% CI: 4.17 to 7.67; I^2^ = 0%) (S12 Fig) and lower preoperative hemoglobin levels (mean difference: -1.29 g/dL; 95% CI: -1.43 to -1.15; I^2^ = 0%) (S13 Fig) than patients without (Table 2).

**Table 2:** Risk factors of postoperative acute myocardial injury.

Risk factors	Number of studies	Number of studies	Effect size	95% CI	Heterogeneity I^2^ (%)
			Mean difference		
Age (years)	6	9314	2.08	-0.47 to 4.62	81%
BMI (kg/m^2^)	5	668	0.35	-0.86 to 1.57	45%
Preoperative hemoglobin (g/dL)	3	8298	-1.29	-1.43 to -1.19	0%
Preoperative hsTnT (ng/L)	3	544	5.92	4.17 to 7.67	0%
			Odds ratio		
Male	6	9327	1.16	0.77 to 1.76	54%
Coronary artery disease	5	9327	2.10	0.85 to 5.21	91%
Hypertension	5	9234	1.47	1.30 to 1.66	0%
Cardiac failure	4	9203	2.63	2.01 to 3.44	15%
Diabetes	4	9203	1.43	1.27 to 1.61	0%
Stroke/TIA	5	9297	0.90	0.50 to 1.59	61%
Renal impairment	5	9297	1.66	1.48 to 1.86	0%
Statins	3	4291	0.65	0.21 to 2.02	89%
Beta-blockers	3	9079	1.65	1.10 to 2.49	48%

BMI: Body Mass Index; TIA: transient ischemic attack; CI: confidence interval; I^2^: the Higgins I-squared index.

### Postoperative acute myocardial injury with postoperative 30-day and long-term mortality

A systematic analysis of the relationship between postoperative myocardial injury and 30-day mortality or long-term mortality was not performed due to insufficient eligible studies. One prospective cohort study conducted by Gualandro et al. [22] reported that the adjusted hazard ratio of POAMI on 30-day mortality is 2.68 (95% CI: 1.85 to 3.86), and the adjusted hazard ratio of POAMI on 1-year mortality is 1.84 (95% CI: 1.52 to 2.24).

### Subgroup analysis

There is no difference in the pooled incidence of POAMI grouped by types of surgeries (*P* = 0.28; I^2^ = 14.6%) (S14 Fig), types of cardiac troponin assays (*P* = 0.29; I^2^ = 11.2%) (S15 Fig), and absolute or relative cardiac troponin changes (*P* = 0.14; I^2^ = 53.3%) (S16 Fig).

### Sensitivity analysis

The definition of POAMI from Chew et al. [26] and Gualandro et al. [22] contains the absolute changes of hsTnT within three days after surgery without considering preoperative hsTnT. After excluding these two studies, the pooled incidence of POAMI is 23% (95% CI: 18% to 27%; I^2^ = 92%).

### Publication bias

A funnel plot was used to assess the publication bias of studies that reported the incidence of POAMI (S17 Fig). Though this funnel plot was visually symmetric, it was hard to rule out the existence of publication bias due to the limited number of studies included for analysis.

## Discussion

This systematic review and meta-analysis revealed that the pooled incidence of POAMI was 20%. Preoperative factors, such as hypertension, diabetes, heart failure, renal impairment, and preoperative beta-blocker intake, were significant risk factors for developing POAMI. In contrast, factors such as sex, body mass index, preoperative coronary artery disease, preoperative stroke/transient ischemic attack, and preoperative statin intake were not associated with POAMI. This systematic analysis identified ten observational studies, which utilized eight different definitions of POAMI. As such, the considerable heterogeneity in the definitions and populations employed across the included studies represents a significant obstacle to accurately assessing the incidence and risk factors of POAMI in noncardiac patients.

The VISION study is a pioneering international prospective cohort investigation that explored myocardial injury in noncardiac patients [3, 13]. By examining the relationship between postoperative myocardial injury and cardiovascular complications and mortality, the VISION study introduced the concept of myocardial injury after noncardiac surgery (MINS) as a diagnostic and prognostic tool, which may facilitate personalized perioperative monitoring and treatment, and postoperative triage. [10]. In the first and second phases of the VISION study, cTnT and hsTnT, respectively, were employed to diagnose MINS [3, 13]. MINS was defined as cTnT≥30 ng/L or hsTnT of 20 to <65 ng/L with an absolute change of at least 5 ng/L, or an hsTnT level ≥65 ng/L, judged as myocardial ischemia within 30 days after noncardiac surgery [3, 13]. MINS does not necessitate the measurement of the patient’s preoperative cTn level, thus lacking the capacity to differentiate acute or chronic myocardial injury. Baseline cTn levels were higher in patients with coronary artery disease, cardiac failure, diabetes, or stroke compared to those without these diseases [12, 18, 40]. Relying solely on postoperative troponin levels for diagnosis of myocardial injury may introduce a potential bias in identifying intraoperative modifiable factors, such as hypotension, that could contribute to myocardial ischemia.

Puelacher et al. employed an hsTnT change of 14 ng/L above preoperative levels to define perioperative myocardial injury (PMI) [14]. The main advantage of this definition is that patients who satisfy it must also meet the criteria for myocardial injury as defined by the Fourth Universal Definition of Myocardial Infarction (2018), which entails a cTn exceeding the 99th percentile URL [9]. The PMI definition may overlook patients with elevated cTn levels of less than 14 ng/L before and after surgery. It remains unclear whether a cTn level difference between 1 and 14 ng/L indicates myocardial injury, or merely reflects a fluctuation in cTn levels due to other factors, such as within-person variation or within-assay variation. Included studies in this review showed a broad range of delta hsTnT, varying from 4 to 40 ng/L in absolute terms and from 20% to 50% in relative terms. Most of these specific delta hsTnT values were derived from previous expert consensus as well as from observational studies. High-grade evidence on the delta cTn for the diagnosis of POAMI is currently lacking. Further studies when applying a change in cTn value to define POAMI may need to clarify reasons for choosing the specific delta value.

Despite the fourth edition of the myocardial infarction guidelines providing a definition for acute myocardial injury, there is currently no consensus on how to accurately measure the extent of cTn changes to predict clinical outcomes [9, 41]. In our analysis of ten studies, there was significant variability in the definitions used to identify POAMI, with eight different definitions being utilized. Based on the fourth definition of myocardial infarction, Chew et al suggested applying a change over three times the standard deviation of the individual variation of a cardiac troponin or 20% elevation from the previous level that exceeds the 99th URL [17]. However, the efficacy of this definition in predicting patient outcomes and guiding interventions requires further investigation through future studies.

To facilitate a more standardized approach, we propose a subdivision of postoperative myocardial injury based on the changes in cTn concentration before and after surgery. This categorization includes three subtypes: first, patients with no myocardial injury before surgery who develop new myocardial injury after surgery; second, patients with chronic myocardial injury before surgery who do not experience new myocardial injury during the perioperative period; and third, patients with chronic myocardial injury before surgery who experience new myocardial injury or aggravation of existing myocardial injury during the perioperative period. This approach may provide clarity and consistency for defining and categorizing postoperative myocardial injury, which may assist future studies to identify intraoperative and postoperative factors leading to myocardial injury.

Preoperative cardiovascular disease is considered a potential risk factor for postoperative cardiovascular complications and mortality [42]. Therefore, current preoperative risk assessments include preexisting cardiovascular complications such as coronary artery disease, diabetes, and stroke [43, 44]. However, our review noticed that preoperative coronary artery disease and stroke were not associated with POAMI. Nevertheless, the definition of POAMI varied across our included studies, and the majority of included studies had a small sample size, which limits the conclusions of risk factors that can be drawn from our systematic review.

This review identified a paucity of studies examining 30-day postoperative and long-term mortality related to POAMI, precluding the ability to conduct a meta-analysis. However, prior observational studies have reported an increased risk of major adverse cardiovascular events, 30-day mortality, and 1-year mortality in patients with postoperative cTn elevation [13, 15, 22, 26, 45]. The detrimental effects of elevated cTn after noncardiac surgery emphasize the significance of early detection and diagnosis of POAMI to facilitate appropriate patient triage and the development of effective monitoring and treatment strategies.

In this review, we assumed that 80% of current studies investigating postoperative myocardial injury in noncardiac patients did not consider the levels of preoperative cTn. Preoperative cTn elevation occurs in about 6% of the population and is even higher in noncardiac patients [12–15]. Only using postoperative cTn concentrations without considering changes in troponin levels before and after surgery to define myocardial injury may not be appropriate to identify intraoperative and postoperative risk factors that damage cardiomyocytes.

The studies included in this review observed a significant elevation of troponin levels occurred in about 20% of patients before and after noncardiac surgery. This result indicates that there are indeed some factors that damage cardiomyocytes during surgery or in the early phase of the postoperative period. While the underlying causes of POAMI remain unclear, current observational studies have suggested that intraoperative and postoperative hypotension, tachycardia, and anemia may disrupt the normal myocardial oxygen supply-demand balance, further promoting myocardial injury [46–49]. Investigational studies aimed at managing these perioperative factors may help elucidate the underlying causes of acute postoperative myocardial injury and guide targeted interventions to enhance patient outcomes following noncardiac surgery.

The present review aims to investigate the potential risk factors contributing to POAMI by examining the existing literature. However, as the included studies in this review were based on observational cohorts, the causality between perioperative risk factors and postoperative AMI remains uncertain. The establishment of sophisticated models via longitudinal analysis of cohort data may prove instrumental in clarifying the causal link between intraoperative and postoperative risk factors and POAMI, thereby providing a theoretical foundation for future interventional randomized controlled trials [50].

It is important to acknowledge several limitations associated with this review. Firstly, the lack of a widely accepted definition of POAMI resulted in significant heterogeneity among the included studies, precluding the accurate estimation of risk factors and outcomes. Additionally, the observational design of the studies, as well as differences in patient characteristics and small sample sizes, may have limited the reliability of the estimates. During the analysis of risk factors for POAMI, various studies have employed different methods to adjust for confounding factors. Consequently, the discrepancies in the adjustments of risk factors across studies may introduce undetectable and unexplainable confounding variables, which can affect the accuracy of the findings. The risk factors summarized in this review should be interpreted with caution. Furthermore, due to the paucity of appropriate studies, the impact of intraoperative and postoperative variables on POAMI was not examined in this review.

## Conclusion

The pooled incidence of POAMI in noncardiac patients was 20% but varied from 10% to 41%. The lack of a universally recognized definition for POAMI, which incorporates diverse cardiac biomarkers and patient groups, poses considerable heterogeneity in accurately analyzing its risk factors. There is a need to establish a uniform definition and cTn measurement procedures based on currently available evidence to facilitate further interventional studies.

## Supporting information

S1 File (PDF)

S1 Checklist PRISMA 2020 checklist. (DOCX)

S1 Table Search strategy. (DOCX)

S2 Table Newcastle-Ottawa Quality Assessment Scale. (DOCX)

S1 Fig Meta-analysis results of the association between preoperative hypertension and the risk of postoperative acute myocardial injury in noncardiac patients. (TIF)

S2 Fig Meta-analysis results of the association between preoperative cardiac failure and the risk of postoperative acute myocardial injury in noncardiac patients. (TIF)

S3 Fig Meta-analysis results of the association between preoperative renal impairment and the risk of postoperative acute myocardial injury in noncardiac patients. (TIF)

S4 Fig Meta-analysis results of the association between preoperative diabetes and the risk of postoperative acute myocardial injury in noncardiac patients. (TIF)

S5 Fig Meta-analysis results of the association between preoperative beta-blocker intake and the risk of postoperative acute myocardial injury in noncardiac patients. (TIF)

S6 Fig Meta-analysis results of the association between age and the risk of postoperative acute myocardial injury in noncardiac patients. (TIF)

S7 Fig Meta-analysis results of the association between male and the risk of postoperative acute myocardial injury in noncardiac patients. (TIF)

S8 Fig Meta-analysis results of the association between BMI and the risk of postoperative acute myocardial injury in noncardiac patients. (TIF)

S9 Fig Meta-analysis results of the association between preoperative coronary artery disease and the risk of postoperative acute myocardial injury in noncardiac patients. (TIF)

S10 Fig Meta-analysis results of the association between preoperative stroke/TIA and the risk of postoperative acute myocardial injury in noncardiac patients. (TIF)

S11 Fig Meta-analysis results of the association between preoperative statins intake and the risk of postoperative acute myocardial injury in noncardiac patients. (TIF)

S12 Fig Meta-analysis results of the association between preoperative hsTnT concentration and the risk of postoperative acute myocardial injury in noncardiac patients. (TIF)

S13 Fig Meta-analysis results of the association between preoperative hemoglobin concentration and the risk of postoperative acute myocardial injury in noncardiac patients. (TIF)

S14 Fig Meta-analysis of postoperative acute myocardial injury in noncardiac patients grouped by types of surgeries. (TIF)

S15 Fig Meta-analysis of postoperative acute myocardial injury in noncardiac patients grouped by types of cardiac troponin assays. (TIF)

S16 Fig Meta-analysis of postoperative acute myocardial injury in noncardiac patients grouped by absolute or relative change before and after surgery. (TIF)

S17 Fig Funnel plot of publication bias of studies reported the incidence of postoperative acute myocardial injury. (TIF)
