# Supplementary material for: Incidence and risk factors of postoperative acute myocardial injury in noncardiac patients: A systematic review and meta-analysis
Source: PLoS One. 2023 Jun 15;18(6):e0286431. doi: 10.1371/journal.pone.0286431 (PMC10270363; doi:10.1371/journal.pone.0286431)
Supplement: S1 File — (PDF) [file pone.0286431.s001.pdf]

Systematic review

*Fields that have an **asterisk (*)** next to them means that they **must be answered. Word limits** are provided for each section. You will be unable to submit the form if the word limits are exceeded for any section. Registrant means the person filling out the form*.

This record cannot be edited because it has been marked as out of scope

1. * Review title.

Give the title of the review in English

Acute Myocardial Injury after Noncardiac Surgery: A Systematic Review and Meta-analysis

2. Original language title.

For reviews in languages other than English, give the title in the original language. This will be displayed with the English language title.

3. * Anticipated or actual start date.

Give the date the systematic review started or is expected to start.

01/02/2023

4. * Anticipated completion date.

Give the date by which the review is expected to be completed.

01/04/2023

5. * Stage of review at time of this submission.

This field uses answers to initial screening questions. It cannot be edited until after registration.

Tick the boxes to show which review tasks have been started and which have been completed.

Update this field each time any amendments are made to a published record.

The review has not yet started: Yes

Review stage	Started	Completed
Preliminary searches	No	No
Piloting of the study selection process	No	No
Formal screening of search results against eligibility criteria	No	No
Data extraction	No	No
Risk of bias (quality) assessment	No	No
Data analysis	No	No

Provide any other relevant information about the stage of the review here.

6. * Named contact.

The named contact is the guarantor for the accuracy of the information in the register record. This may be any member of the review team.

Yuan Chang

Email salutation (e.g. "Dr Smith" or "Joanne") for correspondence:

Dr Chang

7. * Named contact email.

Give the electronic email address of the named contact.

y.chang_@hotmail.com

8. Named contact address

Give the full institutional/organisational postal address for the named contact.

Department of Anesthesiology, First Affiliated Hospital, Kunming Medical University, Kunming City, 650032,

People's Republic of China

9. Named contact phone number.

Give the telephone number for the named contact, including international dialling code.

*8618687506613

10. * Organisational affiliation of the review.

Full title of the organisational affiliations for this review and website address if available. This field may be completed as 'None' if the review is not affiliated to any organisation.

Department of Anesthesiology, First Affiliated Hospital, Kunming Medical University

Organisation web address:

11. * Review team members and their organisational affiliations.

Give the personal details and the organisational affiliations of each member of the review team. Affiliation refers to groups or organisations to which review team members belong. **NOTE: email and country now MUST be entered for each person, unless you are amending a published record.**

Dr Yuan Chang. Department of Anesthesiology, First Affiliated Hospital, Kunming Medical University
Professor Jianlin Shao. Department of Anesthesiology, First Affiliated Hospital, Kunming Medical University
Dr Jing Huang. Department of Anesthesiology, First Affiliated Hospital, Kunming Medical University
Dr Mengjiao Zhou. Department of Anesthesiology, First Affiliated Hospital, Kunming Medical University

12. * Funding sources/sponsors.

Details of the individuals, organizations, groups, companies or other legal entities who have funded or sponsored the review.

None.

Grant number(s)

State the funder, grant or award number and the date of award

None.

13. * Conflicts of interest.

List actual or perceived conflicts of interest (financial or academic).

None

14. Collaborators.

Give the name and affiliation of any individuals or organisations who are working on the review but who are not listed as review team members. **NOTE: email and country must be completed for each person, unless you are amending a published record.**

15. * Review question.

State the review question(s) clearly and precisely. It may be appropriate to break very broad questions down into a series of related more specific questions. Questions may be framed or refined using PI(E)COS or similar where relevant.

Myocardial injury is common after nonsurgery and is associated with significant morbidity and mortality. Myocardial injury is defined as a value of cardiac biomarkers (such as troponin) over the 99th percentile of the upper reference limit of the healthy population. An acute state of myocardial injury is defined as a rise and/or fall in cardiac biomarkers (such as troponin) with at least one value above the 99th percentile upper reference limit. Preoperative chronic cardiac troponin elevation is common. The current definition of postoperative myocardial injury may not distinguish acute myocardial injury from chronic myocardial injury. The incidence, risk factors, and outcomes of postoperative acute myocardial injury are unknown. This meta-analysis aims to synthesize the available evidence on the incidence, risk factors, and outcomes of acute myocardial injury after noncardiac surgery.

16. * Searches.

State the sources that will be searched (e.g. Medline). Give the search dates, and any restrictions (e.g. language or publication date). Do NOT enter the full search strategy (it may be provided as a link or attachment below.)

A comprehensive and systematic literature search was performed utilizing multiple databases including PubMed, Web of Science, and Cochrane CENTRAL to identify relevant studies published prior to February 1st, 2023. The search was performed using the key terms of "troponin," "myocardial injury," "perioperative," and "postoperative," and was limited to English language publications. Two independent researchers carried out the search process, with any discrepancies resolved through a group meeting with a third investigator. To ensure completeness, the reference lists of the included studies were meticulously reviewed and a citation search was also conducted on all eligible studies.

17. URL to search strategy.

Upload a file with your search strategy, or an example of a search strategy for a specific database, (including the keywords) in pdf or word format. In doing so you are consenting to the file being made publicly accessible. Or provide a URL or link to the strategy. Do NOT provide links to your search **results**.

https://www.crd.york.ac.uk/PROSPEROFILES/401607_STRATEGY_20230221.pdf

Alternatively, upload your search strategy to CRD in pdf format. Please note that by doing so you are consenting to the file being made publicly accessible.

Do not make this file publicly available until the review is complete

18. * Condition or domain being studied.

Give a short description of the disease, condition or healthcare domain being studied in your systematic review.

Acute myocardial injury

19. * Participants/population.

Specify the participants or populations being studied in the review. The preferred format includes details of both inclusion and exclusion criteria.

Inclusion: Adult patients underwent noncardiac surgery with preoperative and postoperative cardiac troponin measurements.

Exclusion: Patients under 18 years old; patients undergoing open or interventional cardiac surgery; patients without preoperative cardiac troponin results.

20. * Intervention(s), exposure(s).

Give full and clear descriptions or definitions of the interventions or the exposures to be reviewed. The preferred format includes details of both inclusion and exclusion criteria.

Patients with acute myocardial injury within 30 days after noncardiac surgery. The acute state of myocardial injury should be clearly reported by absolute or relative changes in cardiac troponins before and after surgery.

21. * Comparator(s)/control.

Where relevant, give details of the alternatives against which the intervention/exposure will be compared (e.g. another intervention or a non-exposed control group). The preferred format includes details of both inclusion and exclusion criteria.

Patients without acute myocardial injury within 30 days after noncardiac surgery.

22. * Types of study to be included.

Give details of the study designs (e.g. RCT) that are eligible for inclusion in the review. The preferred format includes both inclusion and exclusion criteria. If there are no restrictions on the types of study, this should be stated.

The inclusion criteria for this study included randomized controlled trials (RCTs) or observational studies (retrospective or prospective), that evaluated changes in troponin levels before and within 30 days after noncardiac surgery in adults who were at least 18 years of age. Exclusion criteria were the following: 1) articles written in languages other than English; 2) reviews, comments, protocols, editorials, letters, case reports, or animal trials; 3) studies with cardiac surgeries; 4) studies used other cardiac biomarkers than cardiac troponins to define myocardial injury; and 5) series with sample sizes of 20. In cases where multiple studies with similar patient populations were identified, the most recent study was selected for inclusion.

23. Context.

Give summary details of the setting or other relevant characteristics, which help define the inclusion or exclusion criteria.

None

24. * Main outcome(s).

Give the pre-specified main (most important) outcomes of the review, including details of how the outcome is defined and measured and when these measurement are made, if these are part of the review inclusion

criteria.

The pooled incidence of acute myocardial injury after noncardiac surgery.

Measures of effect

Please specify the effect measure(s) for you main outcome(s) e.g. relative risks, odds ratios, risk difference, and/or 'number needed to treat.

Absolute events numbers.

25. * Additional outcome(s).

List the pre-specified additional outcomes of the review, with a similar level of detail to that required for main outcomes. Where there are no additional outcomes please state 'None' or 'Not applicable' as appropriate to the review

Risk factors, and 30-day and long-term mortality of acute myocardial injury after noncardiac surgery.

Measures of effect

Please specify the effect measure(s) for you additional outcome(s) e.g. relative risks, odds ratios, risk difference, and/or 'number needed to treat.

Absolute events numbers, odds ratios, and hazard ratios.

26. * Data extraction (selection and coding).

Describe how studies will be selected for inclusion. State what data will be extracted or obtained. State how this will be done and recorded.

The following information will be extracted from each study: name of the first author, year of publication, country, the definition of postoperative myocardial injury, number of patients with and without postoperative myocardial injury, preoperative patient characteristics, and postoperative adverse outcomes. For studies that reported the results of both unpaired and paired cohorts, we will use the unpaired results for the calculation of incidence and risk factors for postoperative acute myocardial injury, and its effect on postoperative death and adverse complications.

27. * Risk of bias (quality) assessment.

State which characteristics of the studies will be assessed and/or any formal risk of bias/quality assessment tools that will be used.

The quality and potential risk of bias of the observational studies will be evaluated using the Newcastle-Ottawa Quality Assessment Scale (NOS) by two investigators. The NOS consists of three dimensions: selection, comparability, and exposure, each of which is assessed by 8 items. A score of 4 points can be assigned for selection, 2 points for comparability, and 3 points for exposure, with a total possible score of 9 points. Higher scores indicate higher quality studies. Studies with scores ranging from 1 to 3 will be low quality, those with scores between 4 and 6 will be intermediate quality, and those with scores greater than 7

will be high quality. The Cochrane Collaboration risk-of-bias tool will assess bias in RCTs in the domains of random sequence generation, allocation concealment, blinding of participants, blinding of out- come assessment, incomplete outcome data, and selective reporting.

28. * Strategy for data synthesis.

Describe the methods you plan to use to synthesise data. This **must not be generic text** but should be **specific to your review** and describe how the proposed approach will be applied to your data. If meta-analysis is planned, describe the models to be used, methods to explore statistical heterogeneity, and software package to be used.

The present study will conduct a meta-analysis using Review Manager 5.3 (Cochrane Collaboration, Oxford, UK). The summary estimates of the included studies will be computed using the inverse variance method with a random-effects model (DerSimonian-Laird estimator) and presented with 95% confidence intervals. Forest plots will be utilized to display the results visually, and funnel plots will be employed to assess publication bias if more than ten studies reported on the same outcome. The presence of statistical heterogeneity will be evaluated through the use of the Cochran Q statistic and quantified by the I^2 statistic.

The incidence rate of postoperative acute myocardial injury will be estimated by pooling the incidence rates and associated 95% confidence intervals from the studies. Continuous and dichotomous preoperative risk factors will be compared using the weighted mean differences and odds ratio (OR), respectively. Risk factors reported by at least three studies will be included. The effect of each included study on 30-day and long-term mortality was assessed using hazard ratio (HR), where long-term mortality is defined as death occurring after 6 months following non-cardiac surgery. The adjusted hazard ratios reported in the included studies will be extracted, and if only unadjusted hazard ratios are available, they will be also extracted and a sensitivity analysis will be conducted to exclude them.

Additionally, sensitivity analyses will determine the robustness of the results by excluding studies that defined postoperative myocardial injury without consideration of the 99th URL of troponin assays, studies that utilized the absolute or relative troponin changes within postoperative days as the definition of postoperative myocardial injury, and studies that were not adjusted for baseline differences by multivariable regression models.

29. * Analysis of subgroups or subsets.

State any planned investigation of ‘subgroups’. Be clear and specific about which type of study or participant will be included in each group or covariate investigated. State the planned analytic approach.

Subgroup analyses will explore the effects of different surgeries and different troponin assays on the pooled incidence of postoperative acute myocardial injury and the hazard ratio of 30-day and long-term mortality.

30. * Type and method of review.

Select the type of review, review method and health area from the lists below.

Type of review

Cost effectiveness

No

Diagnostic

Yes

Epidemiologic

No

Individual patient data (IPD) meta-analysis

No

Intervention

No

Living systematic review

No

Meta-analysis

Yes

Methodology

No

Narrative synthesis

No

Network meta-analysis

No

Pre-clinical

No

Prevention

No

Prognostic

Yes

Prospective meta-analysis (PMA)

No

Review of reviews

No

Service delivery

No

Synthesis of qualitative studies

No

Systematic review

Yes

Other

No

Health area of the review

Alcohol/substance misuse/abuse

No

Blood and immune system

No

Cancer

No

Cardiovascular

Yes

Care of the elderly

No

Child health

No

Complementary therapies

No

COVID-19

No

Crime and justice

No

Dental

No

Digestive system

No

Ear, nose and throat

No

Education

No

Endocrine and metabolic disorders

No

Eye disorders

No

General interest

No

Genetics

No

Health inequalities/health equity

No

Infections and infestations

No

International development

No

Mental health and behavioural conditions

No

Musculoskeletal

No

Neurological

No

Nursing

No

Obstetrics and gynaecology

No

Oral health

No

Palliative care

No

Perioperative care

No

Physiotherapy

No

Pregnancy and childbirth

No

Public health (including social determinants of health)

No

Rehabilitation

No

Respiratory disorders

No

Service delivery

No

Skin disorders

No

Social care

No

Surgery

Yes

Tropical Medicine

No

Urological

No

Wounds, injuries and accidents

No

Violence and abuse

No

31. Language.

Select each language individually to add it to the list below, use the bin icon to remove any added in error.

English

There is not an English language summary

32. * Country.

Select the country in which the review is being carried out. For multi-national collaborations select all the countries involved.

China

33. Other registration details.

Name any other organisation where the systematic review title or protocol is registered (e.g. Campbell, or The Joanna Briggs Institute) together with any unique identification number assigned by them. If extracted data will be stored and made available through a repository such as the Systematic Review Data Repository (SRDR), details and a link should be included here. If none, leave blank.

None.

34. Reference and/or URL for published protocol.

If the protocol for this review is published provide details (authors, title and journal details, preferably in Vancouver format)

Add web link to the published protocol.

Or, upload your published protocol here in pdf format. Note that the upload will be publicly accessible.

No I do not make this file publicly available until the review is complete

Please note that the information required in the PROSPERO registration form must be completed in full even if access to a protocol is given.

35. Dissemination plans.

Do you intend to publish the review on completion?

Yes

Give brief details of plans for communicating review findings.?

36. Keywords.

Give words or phrases that best describe the review. Separate keywords with a semicolon or new line. Keywords help PROSPERO users find your review (keywords do not appear in the public record but are included in searches). Be as specific and precise as possible. Avoid acronyms and abbreviations unless these are in wide use.

Systematic review; meta-analysis; myocardial injury; troponin; postoperative; noncardiac surgery.

37. Details of any existing review of the same topic by the same authors.

If you are registering an update of an existing review give details of the earlier versions and include a full bibliographic reference, if available.

None.

38. * Current review status.

Update review status when the review is completed and when it is published.New registrations must be ongoing so this field is not editable for initial submission.

Please provide anticipated publication date

Review_Ongoing

39. Any additional information.

Provide any other information relevant to the registration of this review.

None.

40. Details of final report/publication(s) or preprints if available.

Leave empty until publication details are available OR you have a link to a preprint (NOTE: this field is not editable for initial submission). List authors, title and journal details preferably in Vancouver format.

Give the link to the published review or preprint.
