# Supplementary material for: Incidence and risk factors of postoperative acute myocardial injury in noncardiac patients: A systematic review and meta-analysis
Source: PLoS One. 2023 Jun 15;18(6):e0286431. doi: 10.1371/journal.pone.0286431 (PMC10270363; doi:10.1371/journal.pone.0286431)
Supplement: S1 Table — (DOCX) [file pone.0286431.s003.docx]

**S2 Table. Search strategy.**

| Database | Query |
| --- | --- |
| PubMed | To February 01, 2023: 3314 items |
|  | (("Troponin"[MeSH Terms:noexp] OR "Troponin T"[MeSH Terms] OR "Troponin I"[MeSH Terms] OR "Troponin"[Supplementary Concept] OR "myocardial injury"[All Fields] OR "myocardial injuries"[All Fields] OR "MINS"[Title]) AND "humans"[MeSH Terms] AND (("perioperative"[Title/Abstract] OR "peri-operative"[Title/Abstract] OR "postoperative"[Title/Abstract] OR "post-operative"[Title/Abstract] OR "surgery"[Title/Abstract] OR "surgical"[Title/Abstract] OR "surgeries"[Title/Abstract] OR "procedure"[Title/Abstract] OR "procedures"[Title/Abstract]) AND "humans"[MeSH Terms])) AND (humans[Filter]) |
| Web of Science | To February 01, 2023: 9990 items |
|  | #1: ((TS=(troponin )) OR TS=(myocardial injury)) OR TS=(myocardial injuries)  #2: ((((((TS=(perioperative)) OR TS=(peri-operative)) OR TS=(postoperative)) OR TS=(post-operative)) OR TS=(surgery)) OR TS=(surgeries)) OR TS=(surgical)  #3: #1 AND #2 |
