# Supplementary material for: Incidence and risk factors of postoperative acute myocardial injury in noncardiac patients: A systematic review and meta-analysis
Source: PLoS One. 2023 Jun 15;18(6):e0286431. doi: 10.1371/journal.pone.0286431 (PMC10270363; doi:10.1371/journal.pone.0286431)
Supplement: S2 Table — (DOCX) [file pone.0286431.s004.docx]

## S3 Table. Newcastle-Ottawa Quality Assessment Scale.

| Study | Selection | | | | Comparability | | Outcome | | |  |
| --- | --- | --- | --- | --- | --- | --- | --- | --- | --- | --- |
|  | Representativeness of exposed cohort | Selection of non- exposed cohort | Ascertainment of exposure | Demonstration that outcome  of interest was not present at start of study | Adjust for the most important risk factors | Adjust for other risk factors | Assessment of outcome | Follow-up length | Loss to follow-up rate | Total quality score |
| Chew et al., 2022 | 1 | 1 | 1 | 1 | 1 | 1 | 1 | 1 | 0 | 8 |
| Gualandro et al., 2021 | 1 | 1 | 1 | 1 | 1 | 1 | 1 | 1 | 0 | 8 |
| Sanderset al., 2021 | 0 | 1 | 1 | 1 | 1 | 1 | 1 | 1 | 0 | 7 |
| Gillmann et al., 2020 | 0 | 1 | 1 | 1 | 1 | 1 | 1 | 0 | 0 | 6 |
| Kamber et al., 2018 | 0 | 1 | 1 | 1 | 0 | 0 | 1 | 1 | 0 | 5 |
| Szczeklik et al., 2018 | 0 | 1 | 1 | 1 | 1 | 1 | 1 | 1 | 1 | 8 |
| Toda et al., 2018 | 0 | 1 | 1 | 1 | 1 | 1 | 1 | 1 | 0 | 7 |
| Górka et al., 2017 | 0 | 1 | 1 | 1 | 1 | 1 | 1 | 1 | 0 | 7 |
| Thomas et al., 2016 | 0 | 1 | 1 | 1 | 1 | 1 | 1 | 0 | 0 | 6 |
| Alcock et al., 2012 | 0 | 1 | 1 | 1 | 1 | 1 | 1 | 1 | 0 | 7 |
