# Supplementary figures and images for: Incidence and risk factors of postoperative acute myocardial injury in noncardiac patients: A systematic review and meta-analysis
Source: PLoS One. 2023 Jun 15;18(6):e0286431. doi: 10.1371/journal.pone.0286431 (PMC10270363; doi:10.1371/journal.pone.0286431)

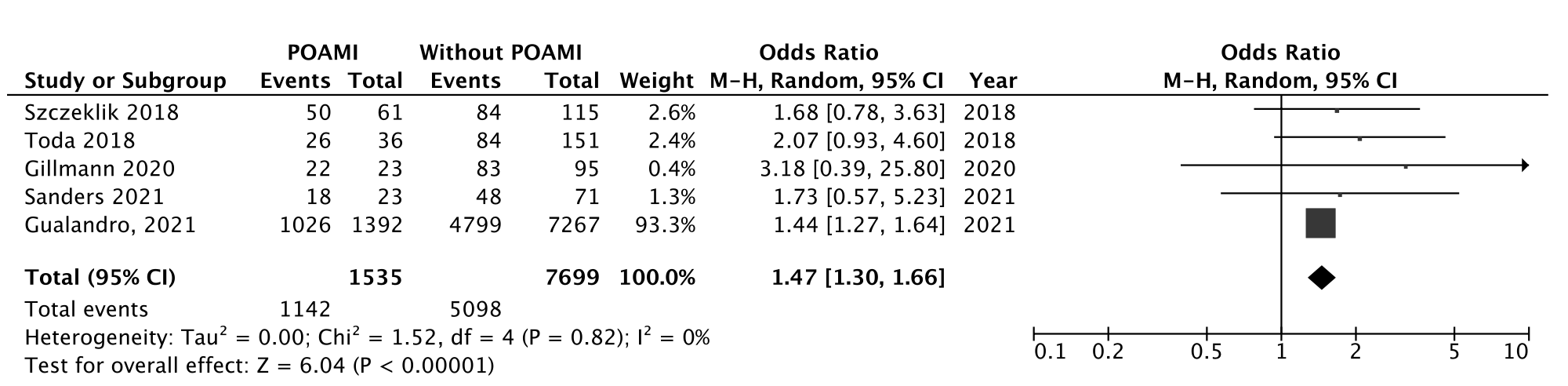

Supplement: S1 Fig — (TIF) [file pone.0286431.s005.tif]

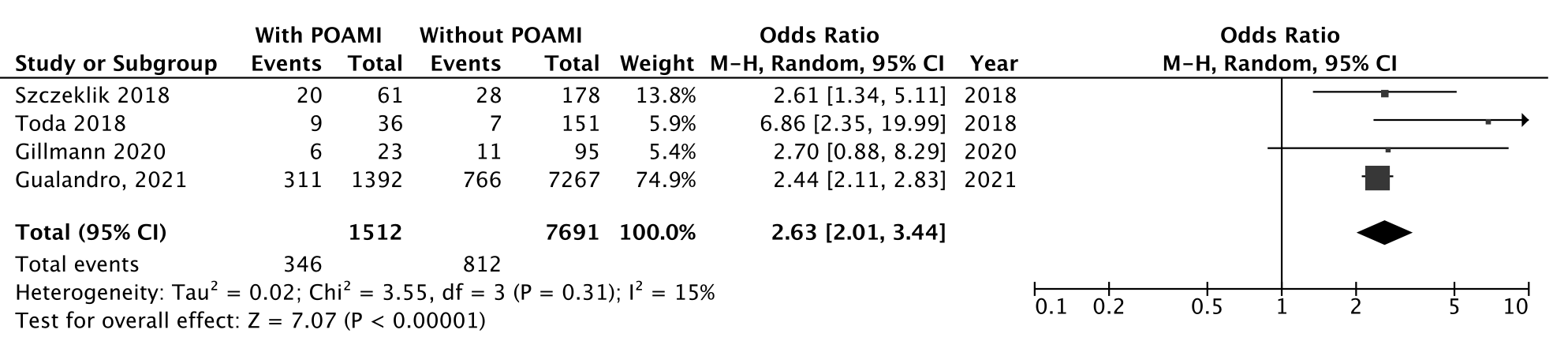

Supplement: S2 Fig — (TIF) [file pone.0286431.s006.tif]

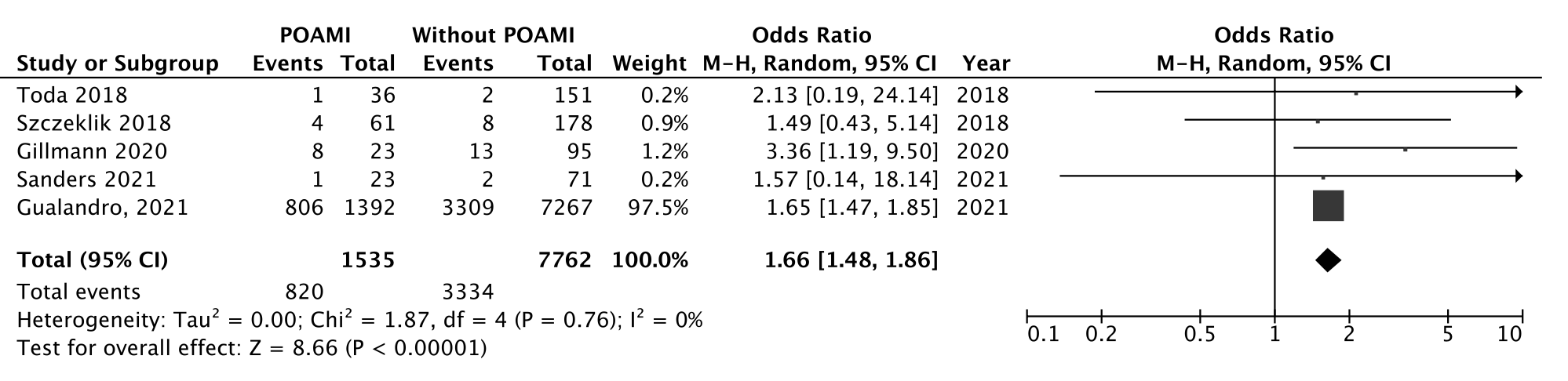

Supplement: S3 Fig — (TIF) [file pone.0286431.s007.tif]

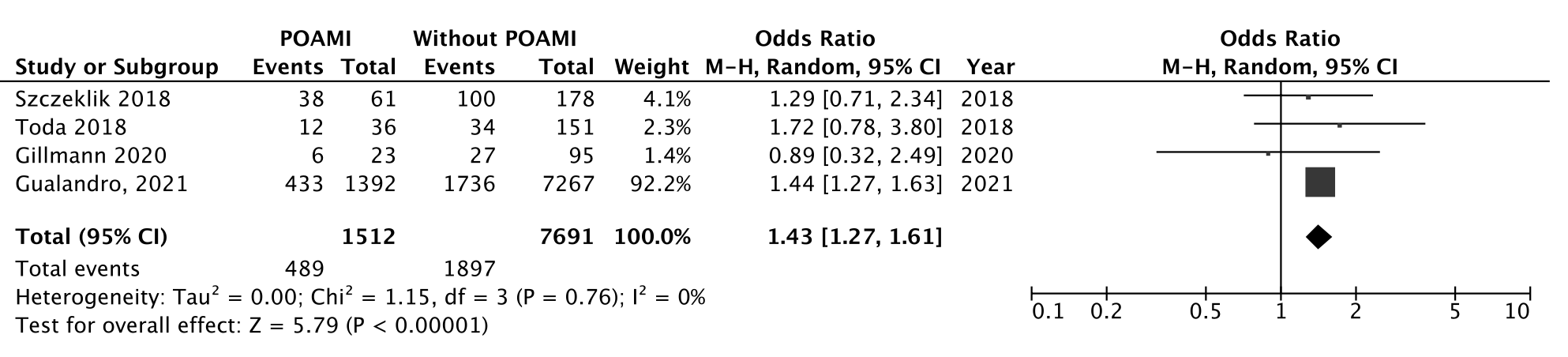

Supplement: S4 Fig — (TIF) [file pone.0286431.s008.tif]

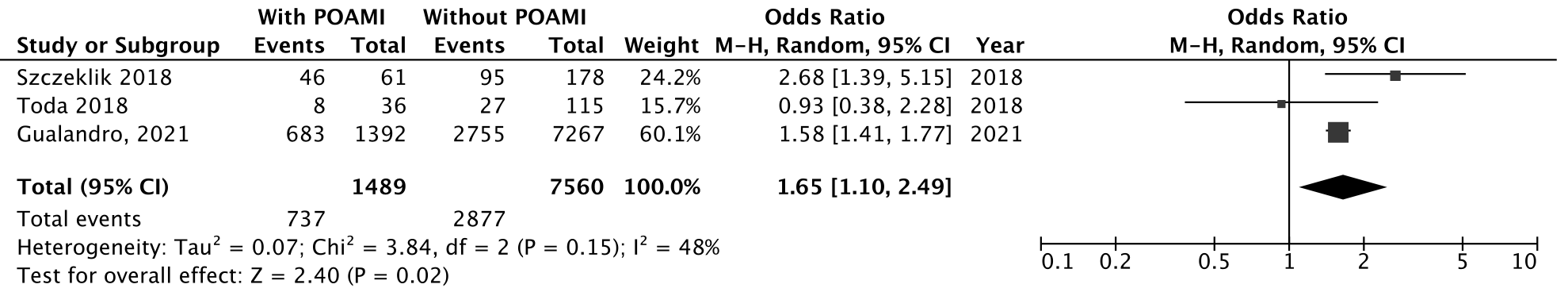

Supplement: S5 Fig — (TIF) [file pone.0286431.s009.tif]

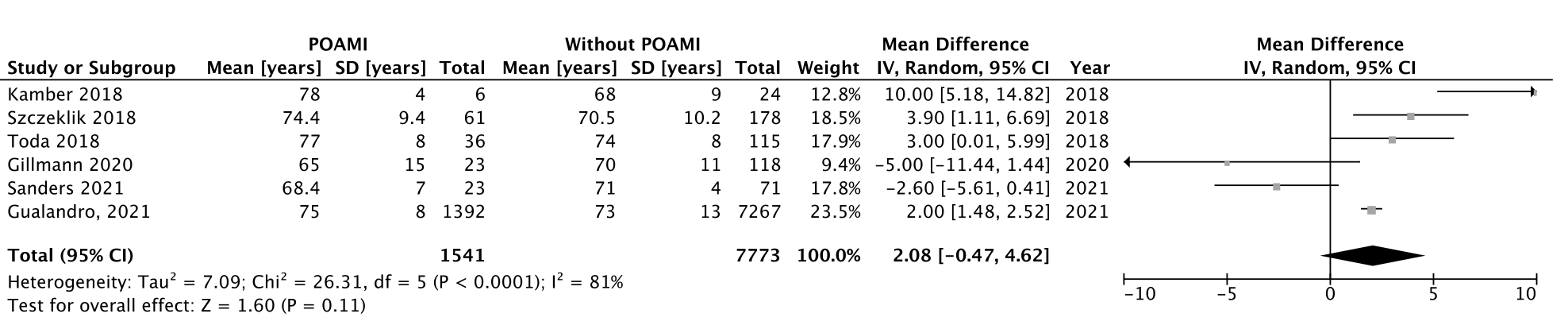

Supplement: S6 Fig — (TIF) [file pone.0286431.s010.tif]

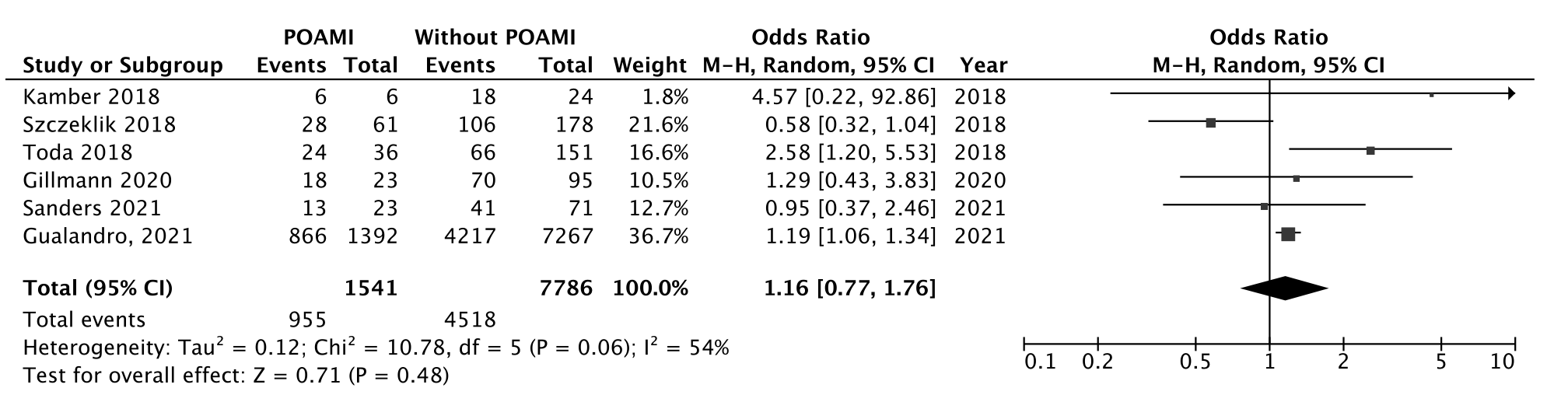

Supplement: S7 Fig — (TIF) [file pone.0286431.s011.tif]

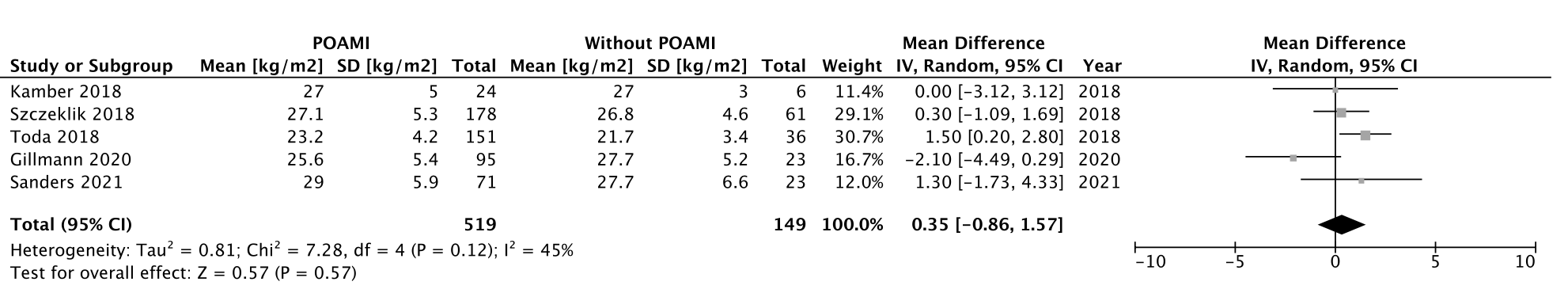

Supplement: S8 Fig — (TIF) [file pone.0286431.s012.tif]

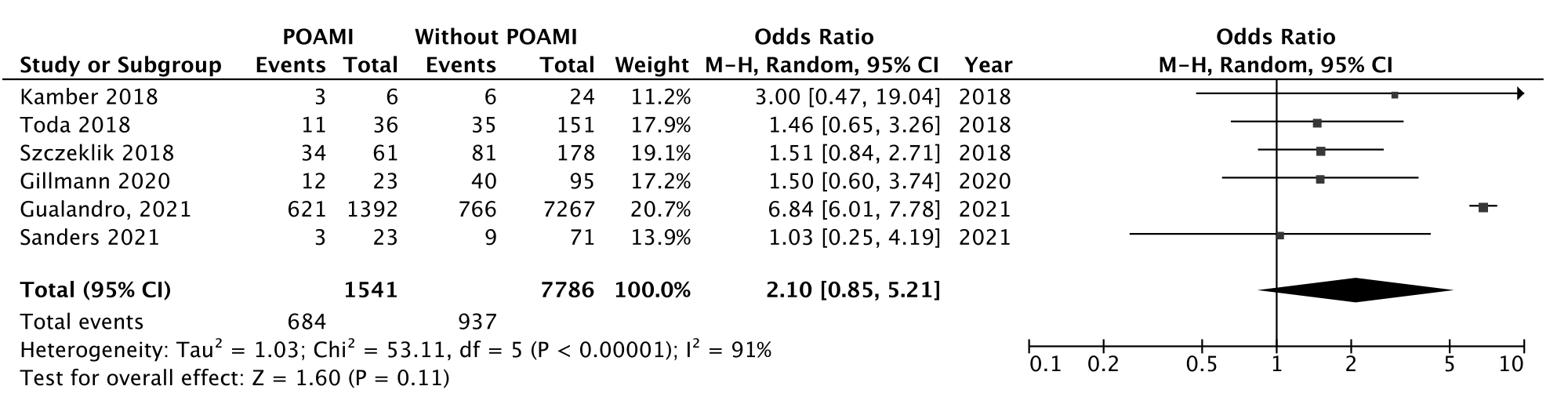

Supplement: S9 Fig — (TIF) [file pone.0286431.s013.tif]

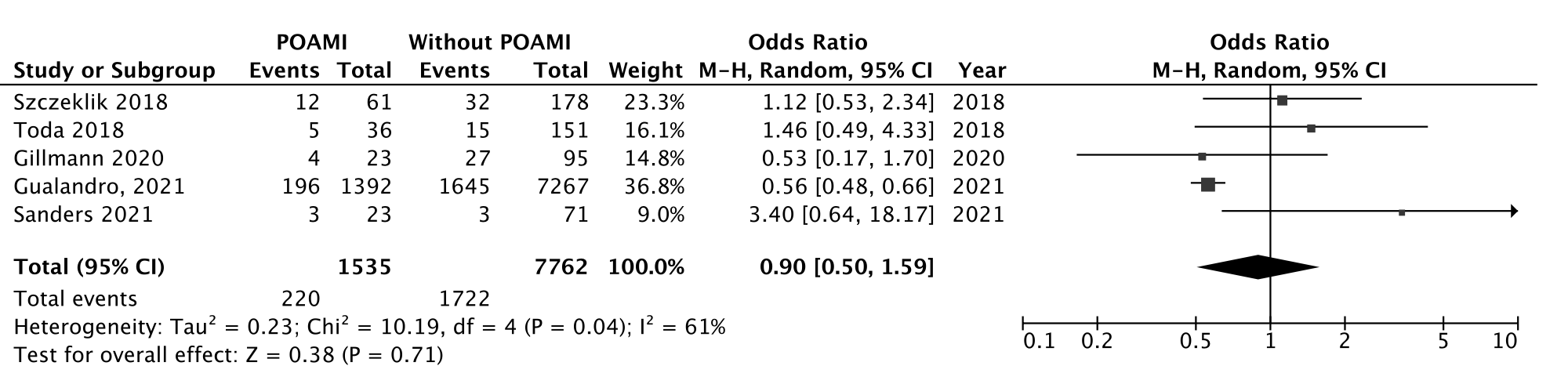

Supplement: S10 Fig — (TIF) [file pone.0286431.s014.tif]

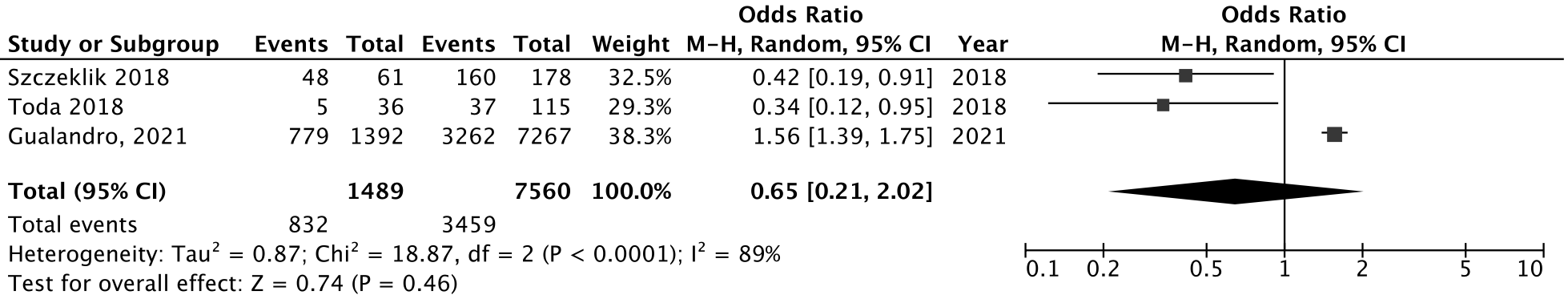

Supplement: S11 Fig — (TIF) [file pone.0286431.s015.tif]

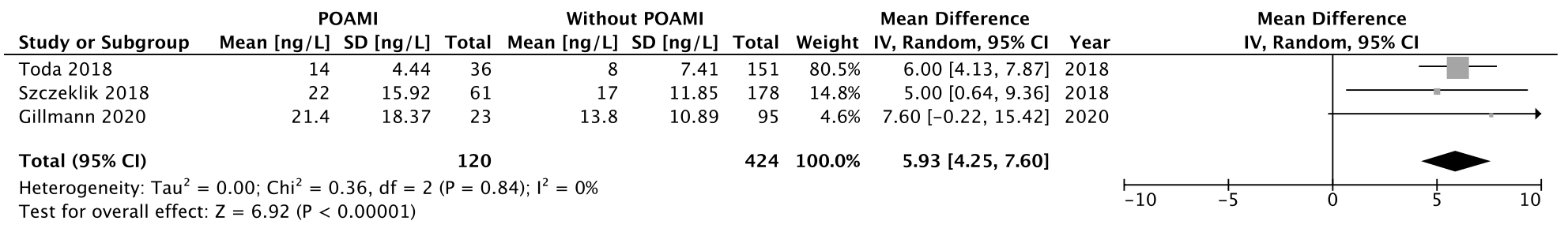

Supplement: S12 Fig — (TIF) [file pone.0286431.s016.tif]

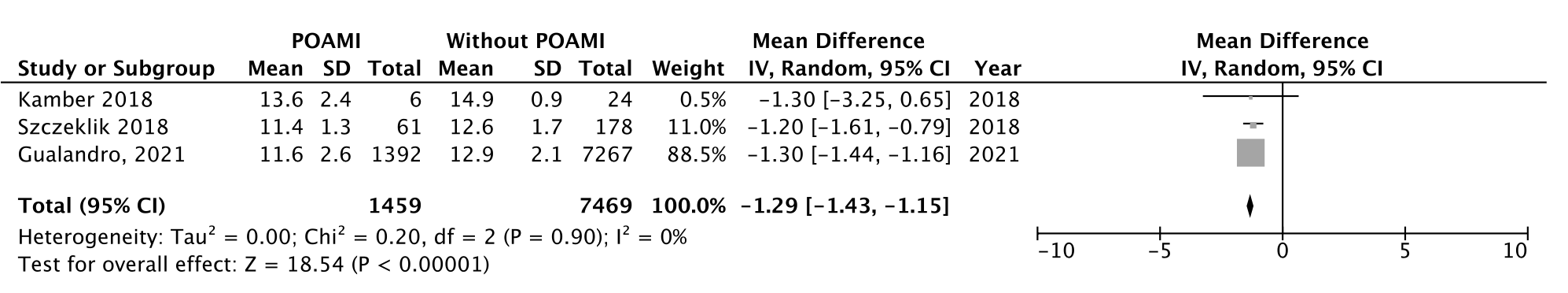

Supplement: S13 Fig — (TIF) [file pone.0286431.s017.tif]

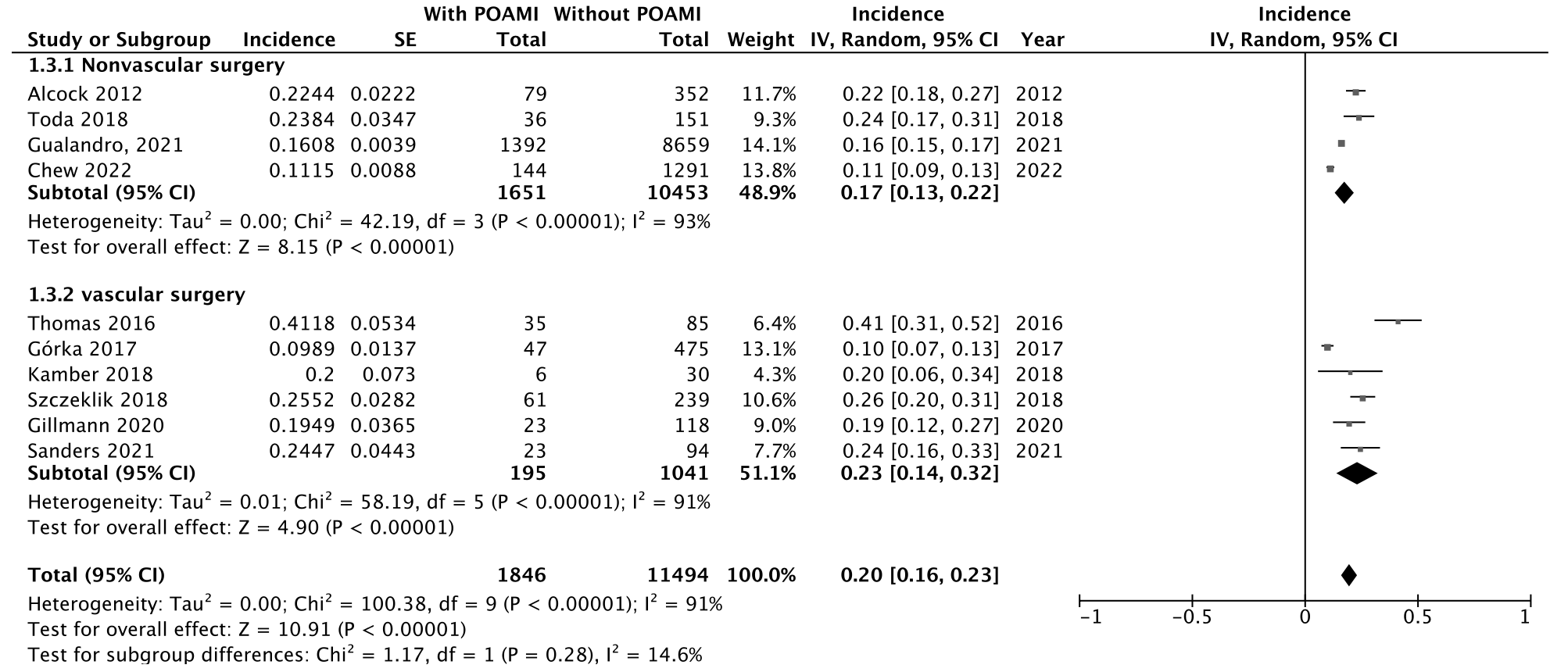

Supplement: S14 Fig — (TIF) [file pone.0286431.s018.tif]

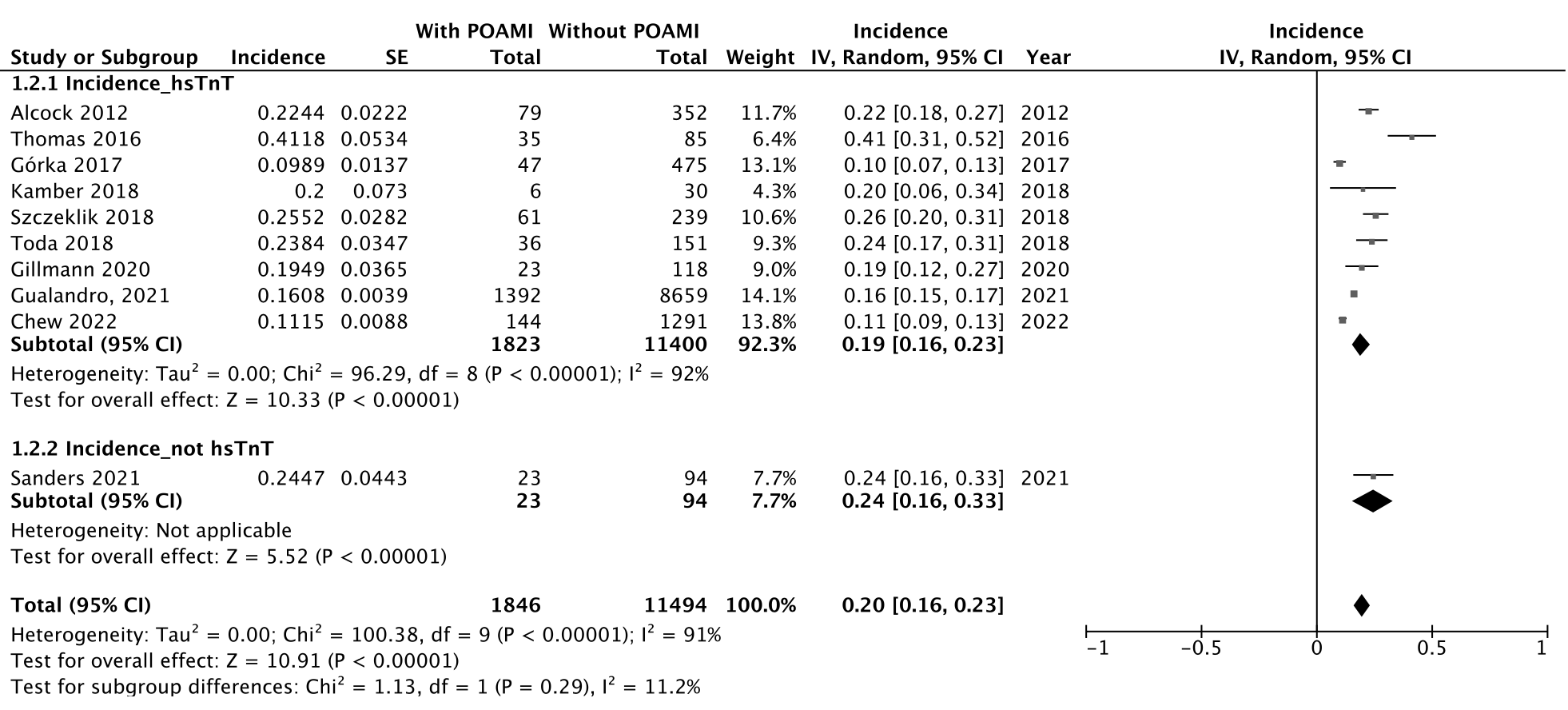

Supplement: S15 Fig — (TIF) [file pone.0286431.s019.tif]

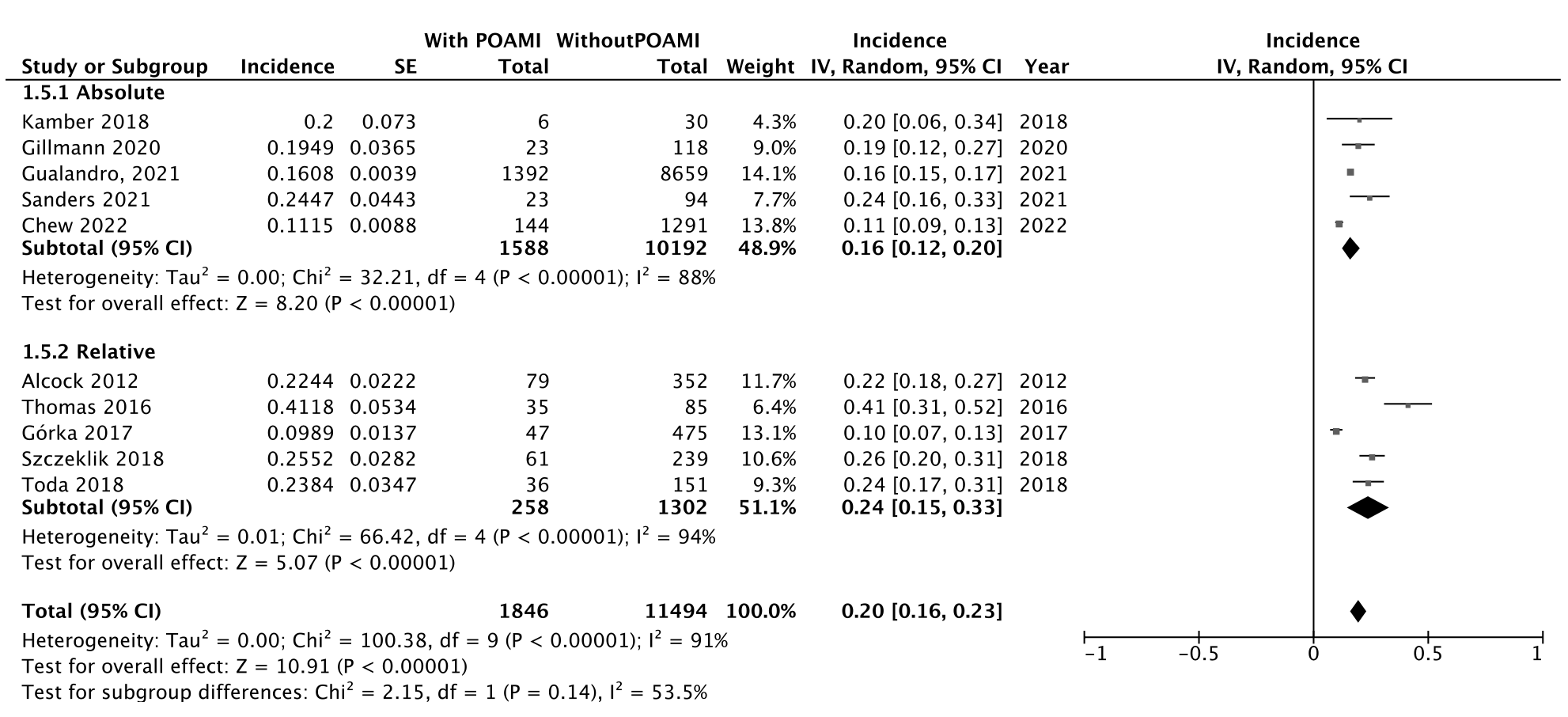

Supplement: S16 Fig — (TIF) [file pone.0286431.s020.tif]

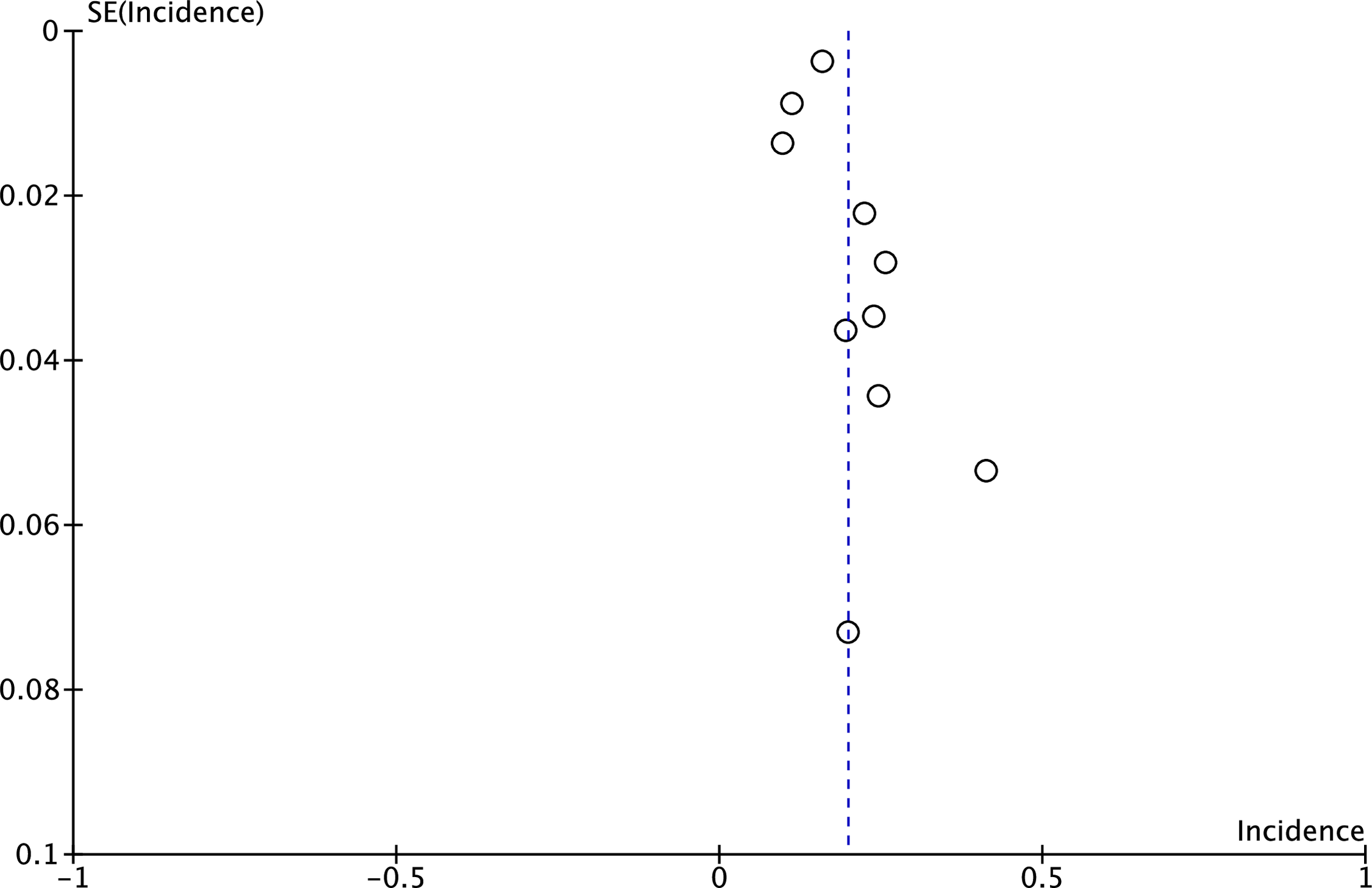

Supplement: S17 Fig — (TIF) [file pone.0286431.s021.tif]
